# Exposures among MERS Case-Patients, Saudi Arabia, January–February 2016

**DOI:** 10.3201/eid2211.161042

**Published:** 2016-11

**Authors:** Raafat F. Alhakeem, Claire M. Midgley, Abdullah M. Assiri, Mohammed Alessa, Hassan Al Hawaj, Abdulaziz Bin Saeed, Malak M. Almasri, Xiaoyan Lu, Glen R. Abedi, Osman Abdalla, Mutaz Mohammed, Homoud S. Algarni, Hail M. Al-Abdely, Ali Abraheem Alsharef, Randa Nooh, Dean D. Erdman, Susan I. Gerber, John T. Watson

**Affiliations:** Ministry of Health, Riyadh, Saudi Arabia (R.F. Alhakeem, A.M. Assiri, M. Alessa, H. Al Hawaj, A. Bin Saeed, M.M. Almasri, O. Abdalla, M. Mohammed, H.S. Algarni, H.M. Al-Abdely, A.A. Alsharef, R. Nooh);; US Centers for Disease Control and Prevention, Atlanta, Georgia, USA (C.M. Midgley, X. Lu, G.R. Abedi, D.D. Erdman. S.I. Gerber, J.T. Watson)

**Keywords:** Middle East respiratory syndrome coronavirus, MERS, MERS-CoV, infections, disease outbreaks, infectious disease transmission, viruses, dromedary, camels, Saudi Arabia, respiratory infections

**To the Editor:** Risk factors for primary acquisition of Middle East respiratory syndrome (MERS) coronavirus (CoV) include recent direct contact with dromedary camels (*1*), but secondary transmission, associated with healthcare settings (*2*–*4*) or household contact (*5*), accounts for most reported cases. Because persons with MERS often do not report any of these risk factors, we investigated MERS cases in Saudi Arabia during an apparent period of limited hospital transmission. Through telephone interviews of case-patients and information from routine investigations, we aimed to characterize exposures and to explore additional factors potentially important in disease transmission. We also genetically sequenced MERS-CoV from respiratory specimens to identify circulating strains.

For confirmed MERS cases (*6*) reported in Saudi Arabia during January–February 2016, we assessed exposures during the 2 weeks before illness onset (exposure period), including direct (*1*) and indirect camel contact; indirect contact was defined as 1) having visited settings where camels were kept but without having direct contact or 2) exposure to friends or household members who themselves had direct camel exposure (*1*). We assessed whether case-patients had worked at, visited, or been admitted to a healthcare setting or had contact with a person known to have MERS during the case-patient’s exposure period. We also asked about recent travel and if any household members were healthcare personnel. For persons too ill to participate or deceased, we interviewed relatives or close friends.

We classified as secondary any case identified through routine case-contact tracing and testing. We considered persons whose cases were identified through routine testing of occupational contacts of MERS-CoV–positive camels to have had direct camel contact. For the remaining cases, we used interview responses to characterize exposures.

All MERS cases reported during January–February 2016 were confirmed in Saudi Arabia by testing of respiratory specimens with real-time reverse transcription PCR assays targeting the MERS-CoV upstream envelope protein gene and the open reading frame (ORF) 1a gene (*7*,*8*). Available specimens (or RNA extracts) were shipped to the US Centers for Disease Control and Prevention (Atlanta, GA, USA) for full genome sequencing. Methods for sequencing and phylogenetic analysis have been described previously (*9*).

During January–February 2016, a total of 27 MERS case-patients were reported by public health officials from 6 of the 13 administrative regions of Saudi Arabia (Technical Appendix 1). Case-patients were evaluated at 20 different hospitals, 3 of which reported >1 case during the investigation period. Among the 27 case-patients, 4 (15%) were identified through routine contact tracing and testing as having secondary cases. Two case-patients (7%) were identified as occupational contacts of MERS-CoV–positive camels. Of the remaining 21 case-patients, 17 (81%) were interviewed during March 13–16, 2016; three were unavailable for interview, and 1 provided incomplete data. Ten (59%) of the 17 interviews were completed by proxy.

Among the 17 case-patients interviewed, 5 (29%) reported direct camel contact (1 of these also reported visiting a healthcare facility), and 4 (24%) reported indirect camel contact (2 of these also reported visiting a healthcare facility) during the exposure period (Technical Appendix 1). Three case-patients reported having close acquaintances who regularly interacted with camels but reported they had not seen these acquaintances during the exposure period. One case-patient was the spouse of a healthcare worker employed in a facility with a reported MERS patient during the putative exposure period; the spouse was found to be MERS-CoV–negative by real-time reverse transcription PCR of a respiratory specimen. The 4 remaining cases could not be further characterized.

Viruses from 13 of the 27 case-patients were sequenced, and all belonged to the MERS-CoV recombinant subclade NRC-2015 (*9*), first detected in humans in January 2015; these 13 case-patients were from the Riyadh and Makkah regions (Technical Appendix 1). Full genome sequences were obtained from the specimens of 11 case-patients (Technical Appendix 2. Continued and predominant circulation of NRC-2015 supports increased epidemiologic fitness compared with other clades, as postulated previously (*9*).

A novel nucleotide substitution was identified in the MERS-CoV sequence from 1 case-patient (Technical Appendix 1) at position 337 located in the stop codon of ORF8b (TAA [Stop] >CAA [Gln] = Stop113Q), predicted to yield a 143aa protein versus the 112aa wild-type. ORF8b is an internal ORF overlapped by the nucleocapsid protein gene (*10*); the corresponding substitution in the nucleocapsid protein gene predicts a conserved amino acid change (V178A). The virologic and clinical significance of these findings is unknown.

Since the emergence of MERS-CoV in 2012, virus acquisition has been associated with direct exposure to camels (*1*) and with person-to-person transmission in households and healthcare settings (*2*–*5*); other sources of infection are less clear. Among the patients in our study whose cases were successfully characterized (23/27), 4 had contact with other known case-patients, and 7 reported direct camel contact. Among the remaining 12 case-patients without these risk factors, 7 were identified as having at least some exposure to persons with direct camel contact. Our findings suggest that community and household exposure to persons with direct camel contact might play a role in MERS-CoV acquisition. Further investigation is needed to determine any specific roles of these interactions in MERS-CoV transmission.

Technical Appendix 1 Characteristics of 27 MERS cases-patients, Saudi Arabia, January–February 2016

Technical Appendix 2 Phylogeny of MERS-CoV genome sequences, Saudi Arabia, January–February 2016.
