# Supplementary material for: Exposures among MERS Case-Patients, Saudi Arabia, January–February 2016
Source: Emerg Infect Dis. 2016 Nov;22(11):2020–2. doi: 10.3201/eid2211.161042 (PMC5088020; doi:10.3201/eid2211.161042)
Supplement: Technical Appendix 2 — Phylogeny of MERS-CoV genome sequences, Saudi Arabia, January–February 2016. [file 16-1042-Techapp-s2.pdf]

Article DOI: http://dx.doi.org/10.3201/eid2211.161042

Exposures among MERS Case-Patients, Saudi Arabia, January–February 2016

Technical Appendix 2

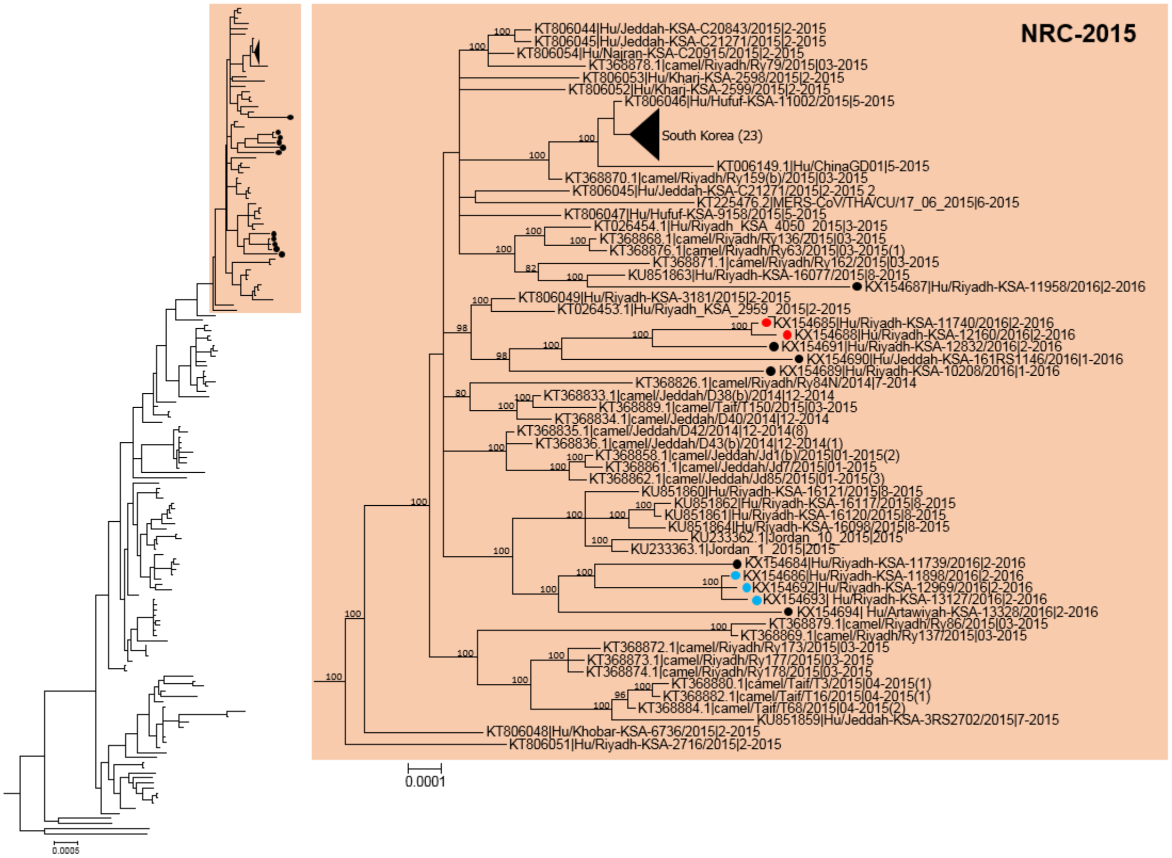

Technical Appendix 2 Figure. Phylogeny of Middle East respiratory syndrome coronavirus (MERS-CoV) genome sequences, Saudi Arabia, January–February 2016. The phylogenetic tree was constructed from MERS-CoV genome sequences obtained from the 11 case-patients reported during January–February 2016, and all near-complete genome sequences available in public domain (n = 213) using the program MrBayes version 3.2.6 under a general time-reversible (GTR) model of nucleotide substitution with 4 categories of γ-distributed rate heterogeneity and a proportion of invariant sites (GTR+4 + I). MERS-CoV sequences belonging to the recently described novel recombinant subclade (NRC-2015) are shown in expanded view. The 11 sequences identified in this study are marked with circles. Red circles denote sequences from the hospitalized case-patient and attending healthcare worker; blue circles denote

sequences from the 3 house-hold contacts. Clade-credibility values $\geq$70% are indicated above the respective nodes. Numbers in brackets following some strain identifiers are the number of additional identical sequences with the same geographic location and sample collection year/month. The scale bar shows the genetic distance as nucleotide substitutions per site.
